# Peripheral inflammation promotes brain tau transmission via disrupting blood–brain barrier

**DOI:** 10.1042/BSR20193629

**Published:** 2020-02-20

**Authors:** Yanchao Liu, Shujuan Zhang, Xiaoguang Li, Enjie Liu, Xin Wang, Qiuzhi Zhou, Jinwang Ye, Jian-Zhi Wang

**Affiliations:** 1 Department of Pathophysiology, School of Basic Medicine, Ministry of Education Key Laboratory for Neurological Disorders, Hubei Key Laboratory for Neurological Disorders, Tongji Medical College, Huazhong University of Science and Technology, Wuhan 430030, China; 2 Co-innovation Center of Neuroregeneration, Nantong University, Nantong 226000, China

**Keywords:** Alzheimers disease, blood brain barrier, lipopolysaccharides, microglia, tau transmission

## Abstract

Abnormal aggregation of pathological tau protein is a neuropathological feature of Alzheimer’s disease (AD). In the AD patients, the abnormal tau accumulation first appeared in entorhinal cortex (EC) and then propagated to the hippocampus with microglia activation and inflammation, but the mechanism is elusive. Here, we studied the role and mechanisms underlying periphery inflammation on brain tau transmission. By intraperitoneal injection of lipopolysaccharide (LPS) with brain medial entorhinal cortex (MEC)-specific overexpressing P301L human tau (P301L-hTau), we found that both acute and chronic administration of LPS remarkably promoted P301L-hTau transmission from MEC to the hippocampal subsets. Interestingly, the chronic LPS-induced P301L-hTau transmission was still apparent after blocking microglia activation. Further studies demonstrated that LPS disrupted the integrity of blood–brain barrier (BBB) and simultaneous intraperitoneal administration of glucocorticoid (GC) attenuated LPS-promoted P301L-hTau transmission. These data together suggest that a non-microglia-dependent BBB disruption contributes to peripheral LPS-promoted brain P301L-hTau transmission, therefore, maintaining the integrity of BBB can be a novel strategy for preventing pathological tau propagation in AD and other tauopathies.

## Introduction

Alzheimer’s disease (AD) is the most common neurodegenerative disease characterized by two major pathological features: senile plaques formed by amyloid β (Aβ) and neurofibrillary tangles formed by pathological tau aggregation [1]. The onset of the disease is concealed, and it can progressively develop into learning and memory dysfunction and even lead to comprehensive dementia. In AD, the irreversibility of the progress and the harmfulness of the results bring a heavy burden to the individual and whole society [2].

As a major microtubule-associated protein (MAP), tau protein plays an important role in promoting microtubule assembly and stabilizing microtubules to maintain normal neuronal morphology [3]. Tau protein, without clear secondary or tertiary structure, is prone to misfolding to form fibrous aggregates under pathological conditions. Studies have shown that pathological tau protein aggregation can spread in different brain regions, which is closely associated with cognitive impairments in AD [4,5]. In the early stage of AD, tau protein first appears in the entorhinal cortex (EC) and then spreads to the hippocampus [6,7]. A recent study has tried to block tau transmission in the P301L mouse model by eliminating microglia and inhibiting the secretion of exosomes [8], but the mechanism for AD-like tau transmission is still unclear.

Lipopolysaccharide (LPS) is a commonly used inflammation inducer, and it is significantly elevated in the hippocampus of AD patients [9]. Many studies have shown that LPS impairs cognitive function in AD mouse model by inducing inflammation, 5-lipoxygenase activation and Aβ production [10–12]. In addition, LPS also causes memory impairment in AD mouse model by activating microglia and disrupting the integrity of the blood−brain barrier (BBB) [13,14]. However, the role of BBB integrity in LPS-induced P301L-hTau transmission has not been reported, to date.

In the present study, we first investigated the effect of acute periphery inflammation induced by intraperitoneal injection of high-dose LPS (10.0 mg/kg for once) [15] on brain tau transmission. We found that the high-dose LPS induced remarkable tau transmission from EC to hippocampal subsets; however, the high-dose LPS caused massive death of mice after 48 h. Considering AD and other tau-related neurodegenerative disorders are generally chronic, therefore, we then investigated whether chronic intraperitoneal injection of lower-dose LPS (0.5 mg/kg twice a week for 5 weeks) could induce brain P301L-hTau transmission and the underlying mechanisms. The results showed that chronic periphery inflammation also promoted brain tau transmission with mechanisms involving a non-microglia-dependent BBB disruption.

## Materials and methods

### Subjects

Male C57BL/6 mice (3 months old) were purchased in Beijing Huafukang Bioscience CO.INC. TLR4tm1.2Karp (null/knockout) mice were purchased in The Jackson Laboratory. All of the mice were kept with plenty of water and food at 23–27°C under a 12-h light/dark cycle. The animal work taken place at the Experimental Animal Center of Tongji Medical College, Huazhong University of Science and Technology. All animal experiments were performed according to the “Policies on the Use of Animals and Humans in Neuroscience Research” by the Society for Neuroscience in 1995 and the ethics approval had been obtained from The Institutional Animal Care and Use Committee at Tongji Medical College, Huazhong University of Science and Technology ([2019] IACUC Number: 2273).

### Reagents and antibodies

The cDNA encoding full-length 441–amino acid human 4-repeat tau bearing a P301L mutation with fusion protein GFP or GFP expression plasmids were cloned into a rAAV9 vector, which had been described previously [16]. Phosphate buffer saline (PBS, Hyclone); lipopolysaccharides (LPS, L2880, Sigma-Aldrich); glucocorticoid (GC, H20051748, Sinopharm).

The primary antibodies used in the present study: GFP (1:500, mouse, ab1218, Abcam); HT7 (1:500, mouse, MN1000, Thermo Fisher); Tau13 (1:500, mouse, sc-21796, Santa Cruz); IBA1 (1:500, goat, ab5076, Abcam); CD68 (1:200, rabbit, A15037, Abclonal); GFAP (1:500, rabbit, ab7260, Abcam); Lectin (1:500, FL1171, Vector Labs); IgG (1:300, A10036, Invitrogen); Hoechst (1:1000, B2261, Sigma-Aldrich); Occludin (1:300, 27260-1-AP, Proteintech), ZO-1 (1:300, 21773-1-AP, Proteintech), and ICAM-1 (1:300, 16174-1-AP, Proteintech).

### Stereotaxic and intraperitoneal injections

The mice were anesthetized with isoflurane and fixed on the stereotactic positioning table. The skin was cleaned and disinfected according to the conventional mouse surgical method. Then, 0.8 μl of viral vectors (10^11^ viral genomes/ml in PBS) were injected into the medial entorhinal cortex (MEC; bregma -4.8 mm, lateral ± 2.9 mm, and depth -3.5 mm) with a Hamilton entry needle. Slow and uniform injection was completed in 10 min, and the needle was taken out after another 10 min. After disinfection and suturing, the mice were placed in a warm and clean cage to wait for recovery, and then returned to the animal house. One week after the stereotactic brain injection, the mice were intraperitoneally injected with PBS (vehicle control) or LPS (0.5 mg/kg) and GC (1.0 mg/kg) twice a week for 5 weeks. To receive better intervention effect, GC was administered 1 h before LPS injection. The behavioral tests were started at day 42 after the brain virus infection with the following sequence: open field to test the motor function at day 43, and then elevated plus arm at day 45, novel object recognition at day 46 and finally Morris water maze from day 48–54. For high-dose LPS [15], single intraperitoneal injection of 10.0 mg/kg LPS was carried out.

### Motor function test

The experimental mice were placed in the test room to familiarize themselves with the environment for 24 h. The experimental box with the upper opening has a specification of 50 cm × 50 cm × 50 cm. At the beginning of the experiment, every mouse was placed in the center of the box bottom with facing straight ahead for 5 min. The Taimeng behavioral detection system (Techman, China) was used to analyze various parameters.

### Elevated plus maze test

The elevated plus maze (EPM) apparatus consisted of four arms (30 cm length × 10 cm width × 15 cm Height) at 90° angles to each other. At the start of a trial, the apparatus was cleaned with 75% alcohol, and the mouse was placed in the center to explore the maze freely for 5 min. The Taimeng behavioral detection system (Techman, China) was used to record and analyze various parameters.

### Novel object recognition

The novel object recognition (NOR) is a cognitive behavioral assay. The test paradigm consists of two phases. In the first phase, all of mice explore two same objects placed diagonally in the box (50 cm × 50 cm × 50 cm) for 5 min. Object exploration was operationally defined as the duration of time mice spent physically contacting the object with a part of the body other than the tail and when mice were facing the object (within 0.5 cm of the object) and actively exploring it (via sniffing or physical manipulation) as previously described [17]. After 24 h, in the second phase, one of the same objects was replaced with a novel object. Record the number of times the mouse explored every object in two phases.

### Morris water maze test

Spatial learning and memory were measured by Morris water maze (MWM) test. The mice were familiar with the environment for 24 h before conducting subsequent experiments. The maze consisted of a circle pool (120 cm in diameter) and a platform (10 cm in diameter and 30 cm in height) placed in the third quadrant. Before the start of the experiment, water keeping at 25 ± 2°C filled into the pool to 1.5 cm above the platform. In spatial learning phase, mice were trained for 3 trials from 2:00 PM to 6:00 PM per day to find the hidden platform for 5 days. Record the time the mice found the platform for a maximum of 60 s and keep them on the platform for 30 s. On the sixth day, the mice were allowed to swim freely in the pool where the platform was removed. All the parameters were recorded by using Noldus video tracking system (Ethovision).

### *In vivo* microdialysis and hTau assay

The mice were anesthetized with 2% isoflurane and subjected to a stereotaxic surgery to insert a cannula into dentate gyrus (DG). The method of microdialysis *in vivo* was described previously [16]. An ELISA kit was applied to measure total hTau present in microdialysis samples according to manufacturer’s instructions (KHB0041, Thermo Fisher).

### Immunohistochemistry and immunofluorescence

Mice were killed after anesthetized with 6% chloral hydrate and perfused by 4% paraformaldehyde. The brains were cut into 30 μm coronal sections by Vibratome (VT1000S, Leica). For immunohistochemistry (IHC) and immunofluorescence (IF), the experiments were carried out according to the established methods [18]. For IHC, the sections were incubated with IBA1 and GFAP (1:500, Abcam) primary antibody overnight at 4°C. The images were acquired with a microscope (Nikon, Japan). For IF, the sections were incubated with primary antibodies (HT7, 1:500, Thermo Fisher; Lectin, 1:500, Vector Labs; IgG, 1:300, Invitrogen) overnight at 4°C. The images were scanned with Carl Zeiss LSM710 confocal microscope, and ImageJ2x 2.1.4.5 was used for the quantitative analysis of the IHC and IF images. For IHC, we used average optical density (AOD, AOD = IOD/area, i.e. the concentration per unit area of the target substance), to evaluate the differences between groups of the brain sections. For IF, we determined the fluorescent intensity based on the cell morphology.

### Statistical analysis

All data were analyzed using Graphpad Prism 8.0. Data were present as mean ± SD or mean ± SEM. Data comparison between the two groups using Student *t* test. The statistical comparison method of data between multiple groups is one-way or two-way ANOVA followed by post hoc tests. *P* < 0.05 was considered statistical significance.

## Results

### Acute high-dose LPS induces P301L-hTau spreading from EC to hippocampus and GC attenuates hTau transmission without affecting microglia activation

**Figure 1:**
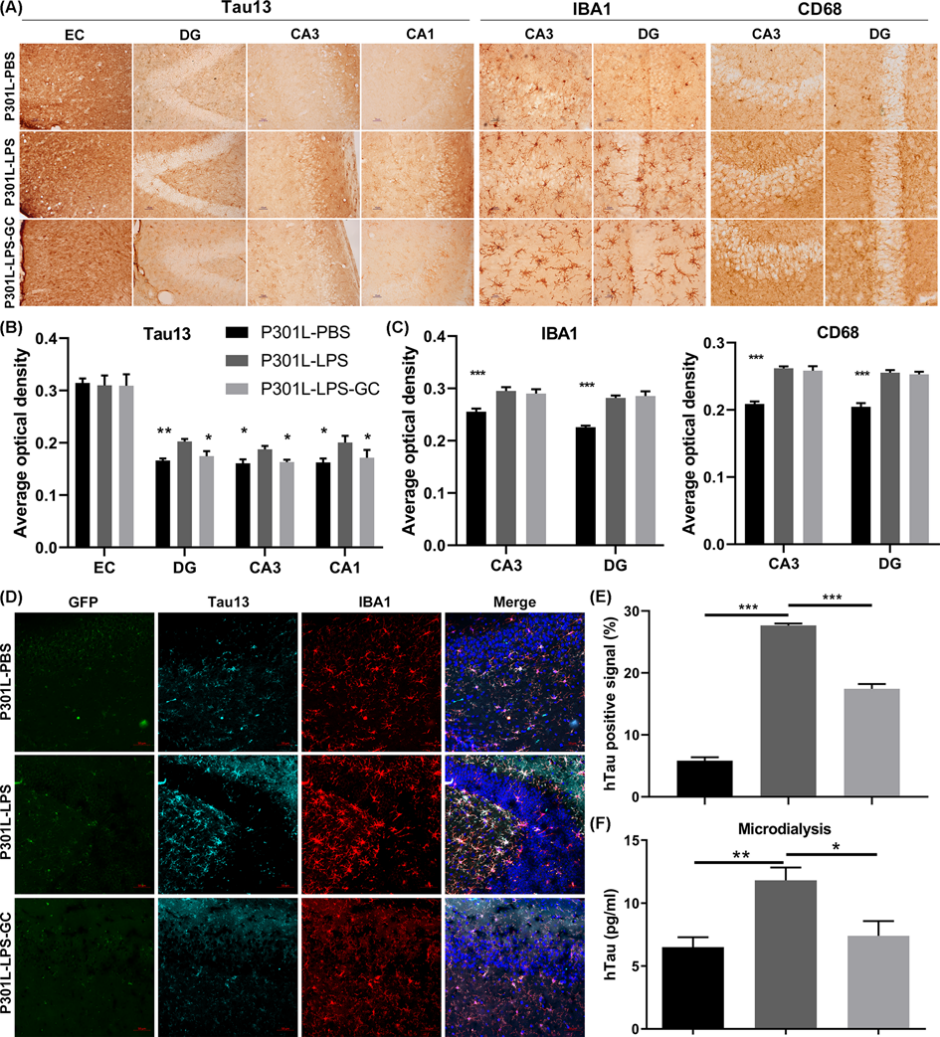
Acute high-dose LPS induces P301L-hTau spreading from EC to hippocampus and GC attenuates hTau transmission without affecting microglia activation (**A**) LPS increased hTau spreading from EC to hippocampal subsets (DG, CA3 and CA1), and GC attenuated hTau transmission (Tau13) without suppression of microglia activation detected by immunohistochemical staining. Tau13 specifically reacts with human tau; scale bar, 10 μm. Average optical density (AOD) was used to estimate the intensity of Tau13 (**B**), IBA1 and CD68 (**C**). Data were present as mean ± SD (*n* = 3 each group, two-way ANOVA followed by Tukey *post hoc* test). *, *P* < 0.05; **, *P* < 0.01; ***, *P* < 0.001 versus P301L-LPS. (**D,E**) LPS-induced hTau transmission was co-localized with the activated microglia in hippocampal DG subset measured by co-immunofluorescence, and GC treatment attenuated hTau transmission without suppression of microglia activation. Data were present as mean ± SD (*n* = 3 each group, one-way ANOVA followed by Tukey *post hoc* test). ***, *P* < 0.001. (**F**) LPS increased hTau level in DG interstitial fluid measured by microdialysis and ELISA, and GC attenuated the elevation of hTau in DG subset; scale bar, 50 μm. Data were present as mean ± SEM (*n* = 6∼8 each group, one-way ANOVA followed by Bonferroni *post hoc* test). *, *P* < 0.05; **, *P* < 0.01.

To explore whether the acute periphery inflammation promote tau transmission in the brain, we infused stereotaxically rAAV-GFP-P301L into the MEC of mice (3 months old), after 7 days, LPS (10.0 mg/kg) or PBS (vehicle control), and GC (20.0 mg/kg) were administrated through intraperitoneal injection. After 24 h, the interstitial fluid at DG subset was collected by microdialysis. During the study, we observed massive death of mice started at 36 h after high-dose LPS injection. Therefore, we killed the mice at 36 h, and measured hTau in EC (injected site) and hippocampus (transmitted from EC) by immunohistochemistry, co-immunofluorescence and ELISA. A remarkably increased Tau13 (specifically reacts with human tau) was detected in hippocampal DG, CA3 and CA1 subsets of P301L-LPS-injected group compared with P301L-PBS group, and GC treatment attenuated hTau transmission (Figure 1A,B) without affected high-dose LPS-induced microglial activation (Figure 1A,C). In P301L-LPS group, an increased hTau co-localized with activated microglia in DG subset was also detected by immunofluorescence staining (Figure 1D,E). By microdialysis and ELISA assay, a remarkably increased hTau level in the hippocampal DG interstitial fluid was shown in of P301L-hTau-LPS and GC treatment reduced the extracellular hTau level at DG (Figure 1F). These data together suggest that LPS accelerates hTau transmission from EC to the hippocampus and GC attenuates LPS-induced hTau transmission.

### Chronic lower-dose LPS enhances P301L-hTau-induced cognitive deficit and GC alleviates the effect of LPS

**Figure 2:**
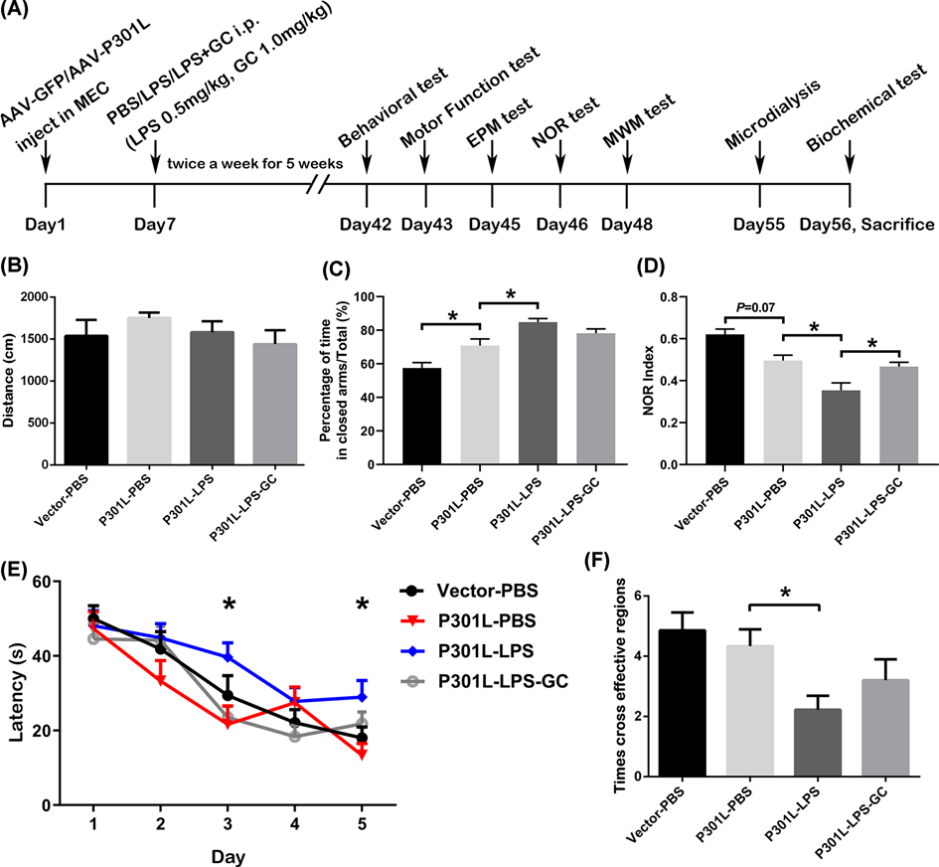
Chronic lower-dose LPS enhances P301L-hTau-induced cognitive impairment and GC partially ameliorates cognitive dysfunction in mice (**A**) Schematic diagram showing experimental procedure: AAV-GFP/P301L-hTau was injected into MEC of 4-month-old C57BL/6 mice. Seven days later, LPS or PBS and GC were injected intraperitoneally twice a week for 5 weeks. Behavioral tests, microdialysis and biochemical assays were performed subsequently. (**B**) No difference in motor function between the four groups of mice was detected. (**C**) In the EPM test, LPS enhanced hTau-induced anxiety-like behavior, which could be partially restored by GC treatment. (**D**) LPS enhanced hTau-induced memory impairment shown by the further decreased NOR Index, and GC ameliorated LPS effect. (**E**) In MWM training, the escape latency of the P301L-LPS mice increased compared with the P301L-PBS mice at day 3 and 5. Time factor, DF = 4, F(4, 448) = 32.4, *P* < 0.0001; treatment factor, DF = 3, F(3, 448) = 5.225, *P* = 0.0015; Interaction, *P* > 0.05. (**F**) LPS enhanced hTau-induced memory impairment shown by the decreased crossing times at target quadrant after removed the platform, GC partially antagonized LPS effect. Data was present as mean ± SEM (*n* = 9–11 each group, one-way ANOVA followed by Bonferroni *post hoc* test (**B,C,D,F**), two-way ANOVA followed by Bonferroni *post hoc* test (**E**)). *, *P* < 0.05.

To explore whether chronic LPS exposure, which mimics the chronic course of AD-like neurodegeneration, can enhance hTau-induced impairments, we infused stereotaxically rAAV-GFP-P301L or its empty vector into the MEC of 3-month-old mice. After 7 days, LPS (0.5 mg/kg) or PBS (vehicle control), and GC (1.0 mg/kg) were simultaneously injected twice a week for five weeks through intraperitoneal. None-selective expression of AAV-hTau was detected in neurons, microglia and astrocytes of the EC subset in PBS-injected group (Supplementary Figure S1). To receive better intervention effect, GC was administered 1 h before LPS injection (Figure 2A). No difference of motor function was detected in the mice after brain surgery and LPS exposure (Figure 2B). By elevated plus maze (EPM) test, we observed that the P301L-hTau mice spent longer time in EPM and LPS further increased the closed arm time in P301L-hTau mice, while GC treatment restored the time in closed arm (Figure 2C). These data suggest that LPS enhances P301L-hTau-induced anxiety-like behavior, while GC intervention alleviates the anxiety-like behavior of the mice.

Then, we examined learning and memory function of the mice by novel object recognition (NOR) and Morris water maze (MWM) tests. Based on the NOR Index (Index = Novel/ (Novel + Old)), we observed that LPS enhanced P301L-hTau-induced reduction of NOR, while GC intervention partially restored the NOR score (Figure 2D). In the MWM test, the LPS treatment further increased the latency time to find the hidden platform on the basis of P301L-hTau group during 5 days training, while GC reduced the latency (Figure 2E). The spatial memory was tested by removed the platform on day 6. The effective cross-time in the platform area was significantly decreased in P301L-hTau/LPS group (Figure 2F). These data together suggest that LPS accelerates P301L-hTau-induced cognitive impairments and GC can partial attenuate the P301L-hTau/LPS-induced cognitive deficits.

### Chronic lower-dose LPS promotes hTau spreading from EC to hippocampus and GC attenuates the spreading

**Figure 3:**
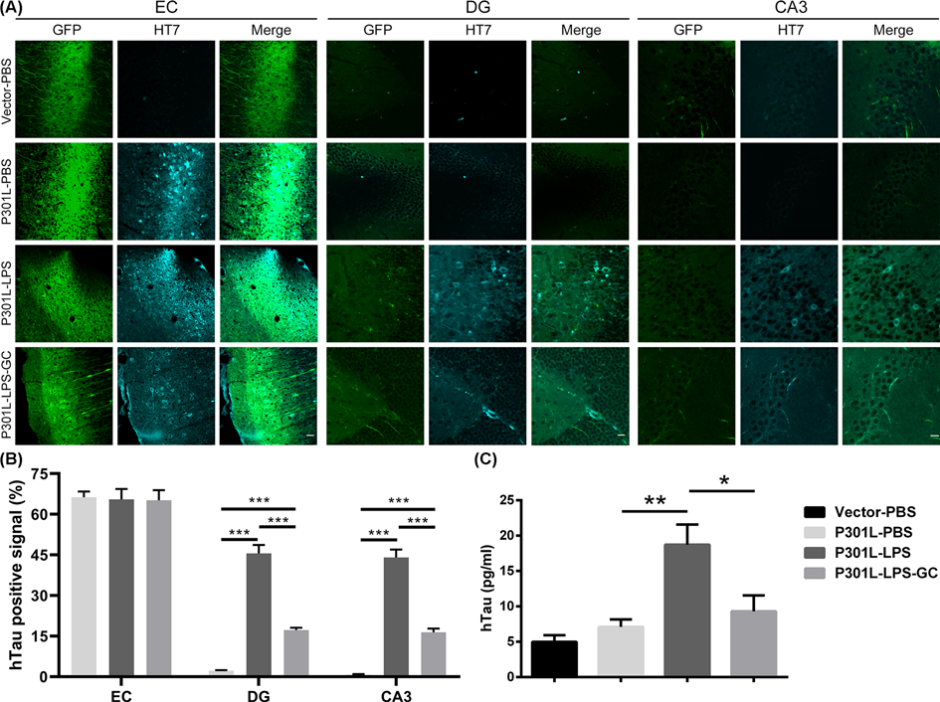
Chronic low-dose LPS induces P301L-hTau spreading from EC to hippocampus and GC attenuates the transmission (**A**) Mice were treated as described in Figure 2. After behavioral tests, the mice were killed and the brain slices were subjected to immunofluorescence for GFP (green) and HT7 (specific to hTau, cyan) in EC, DG and CA3. EC scale bar, 50 μm; DG and CA3, scale bar: 20 μm. (**B**) Quantification of hTau positive signal (HT7) in EC, DG and CA3. Data were presented as mean ± SD (*n* = 3 each group, two-way ANOVA followed by Tukey *post hoc* test). ***, *P* < 0.001. (**C**) The interstitial fluid level of hTau at DG subset was significantly increased in P301L-hTau-LPS group while GC attenuated the elevation of hTau in the interstitial fluid collected by microdialysis and analyzed by ELISA. Data were present as mean ± SEM (*n* = 7∼10 each group, one-way ANOVA followed by Bonferroni *post hoc* test). *, *P* < 0.05. **, *P* < 0.01.

To investigate whether LPS can accelerate hTau transmission from EC to hippocampus, we used HT7 antibody to probe hTau by immunofluorescence. We observed that expressing P30L-hTau alone did not induce significant hTau spreading from EC to DG and CA3, while LPS remarkably enhanced hTau transmission and GC treatment blocked the transmission (Figure 3A,B). By microdialysis at DG subset, we observed that hTau level in the interstitial fluid of P301L-hTau-LPS group was remarkably higher than the other groups, and GC intervention reduced hTau level in the interstitial fluid (Figure 3C). There was no significant difference in hTau expression in the EC between the three P301L groups (Supplementary Figure S2). These data suggest that LPS accelerates hTau transmission from EC to the hippocampus and GC attenuates LPS-induced hTau transmission.

### Chronic low-dose LPS does not increase hTau-induced microglia activation

**Figure 4:**
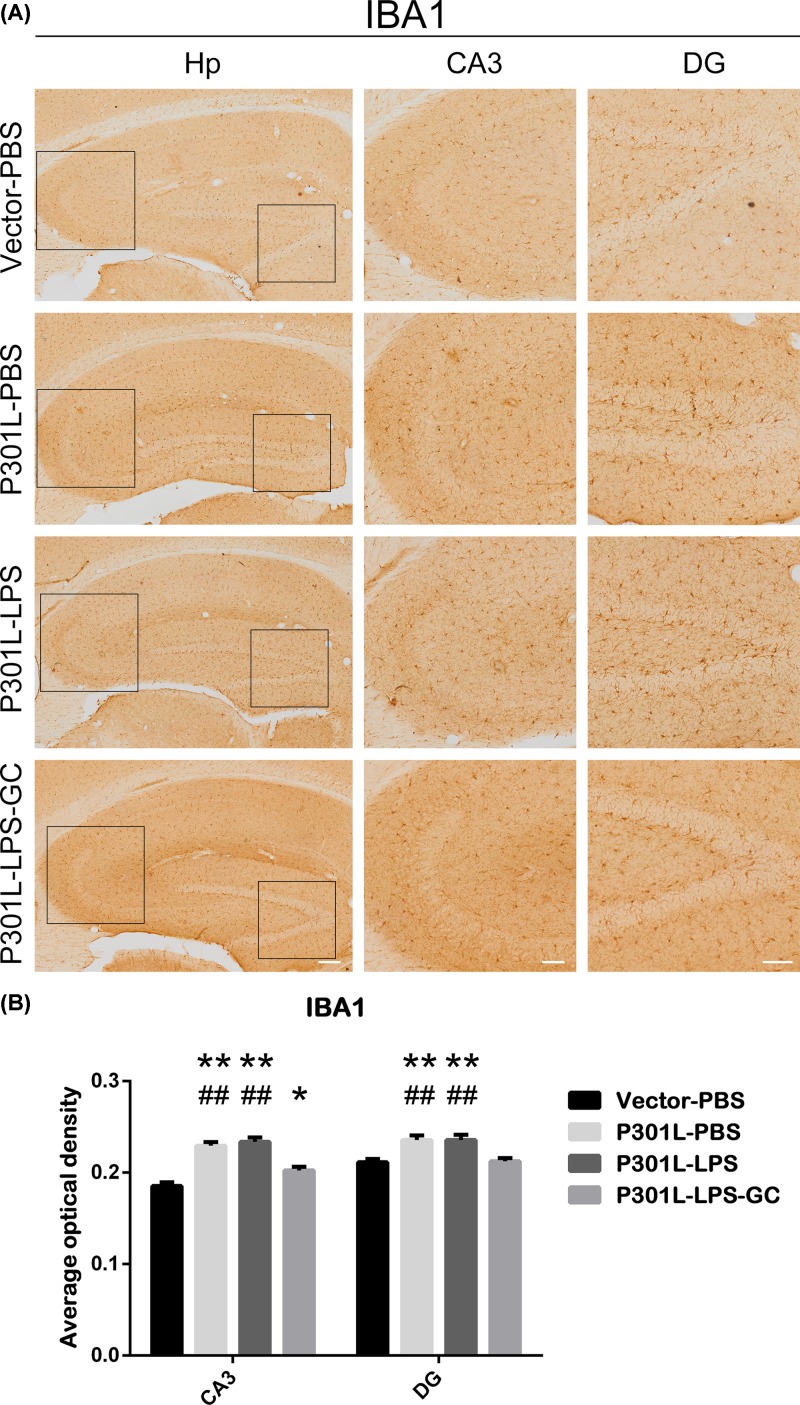
Chronic low-dose LPS does not enhance P301L-mediated microglia activation and GC attenuates hTau-induced activation of microglia in both CA3 and DG subsets Mice were treated as described in Figure 2. (**A**) Activation of microglia (IBA1) was measured by immunohistochemical staining. Hippocampus (Hp) scale bar, 200 μm; CA3 and DG scale bar, 100 μm. (**B**) Average optical density (AOD) was used to analyze the activation of microglia. Data were present as mean ± SD (*n* = 3 each group, two-way ANOVA followed by Tukey *post hoc* test). *, *P* < 0.05; **, *P* < 0.01 versus Vector-PBS. ##, *P* < 0.01 versus P301L-LPS-GC.

To explore microglia involvement in hTau transmission, we observed that microglia (IBA1) was remarkably activated in P301L-hTau group, while no further microglia activation was detected after LPS injection (Figure 4A,B). GC treatment partially attenuated microglial activation in both CA3 and DG subsets (Figure 4A,B). These data suggest that microglial activation only partially involved in hTau transmission.

### Knockout TLR4 attenuates microglial activation without ameliorating LPS-enhanced hTau transmission

**Figure 5:**
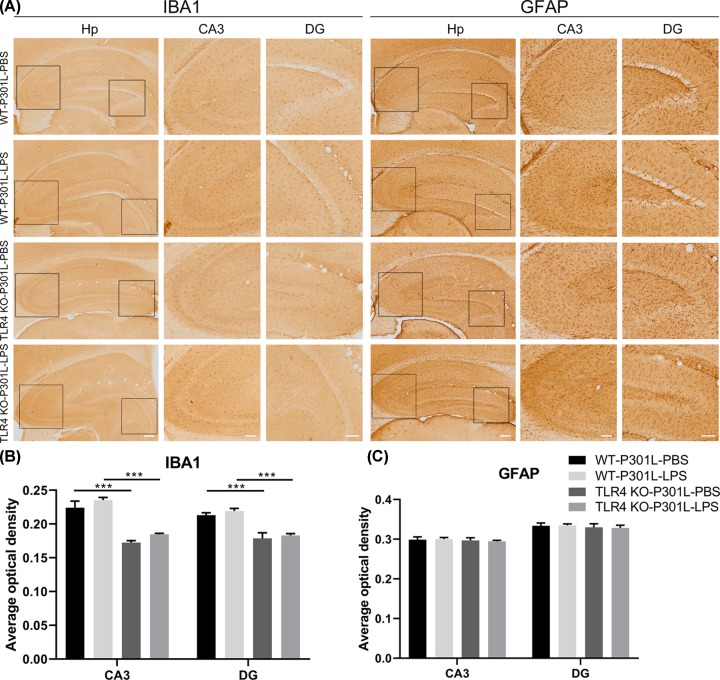
Knockout TLR4 attenuates microglial activation without ameliorating LPS-enhanced hTau transmission (**A**) TLR4 KO attenuates LPS-enhanced microglia activation (IBA1) without affect astrocytes (GFAP) measured by immunohistochemical staining compared with WT-P301L-LPS mice. Hp scale bar, 200 μm; CA3 scale bar, 100 μm; DG scale bar, 100 μm. (**B** and **C**) Average optical density (AOD) was used to analyze the activation of microglia (B) and astrocytes (C). Data were present as mean ± SD (*n* = 3 each group, two-way ANOVA followed by Tukey *post hoc* test). ***, *P* < 0.001 versus WT-P301L-LPS.

A previous study indicated that inhibition of microglia activation could reduce the hTau transmission in P301L mouse model [8]. To explore the mechanisms underlying LPS-mediated hTau transmission, we studied the involvement of Toll like receptor 4 (TLR4), an important receptor required for LPS to activate microglia. AAV-P301L-hTau was expressed in MEC of TLR4 knockout (TLR4 KO) or the wild-type (WT) mice for 7 days, and then injected LPS through intraperitoneal twice a week for 5 weeks. We observed that TLR4 knockout significantly attenuated P301L-hTau-LPS-induced microglia activation without affect astrocytes (GFAP) in hippocampal DG and CA3 subsets when compared with the WT-P301L-LPS group (Figure 5A–C). These data suggest that knockout TLR4 specifically attenuates microglial activation without affecting astrocytes.

**Figure 6:**
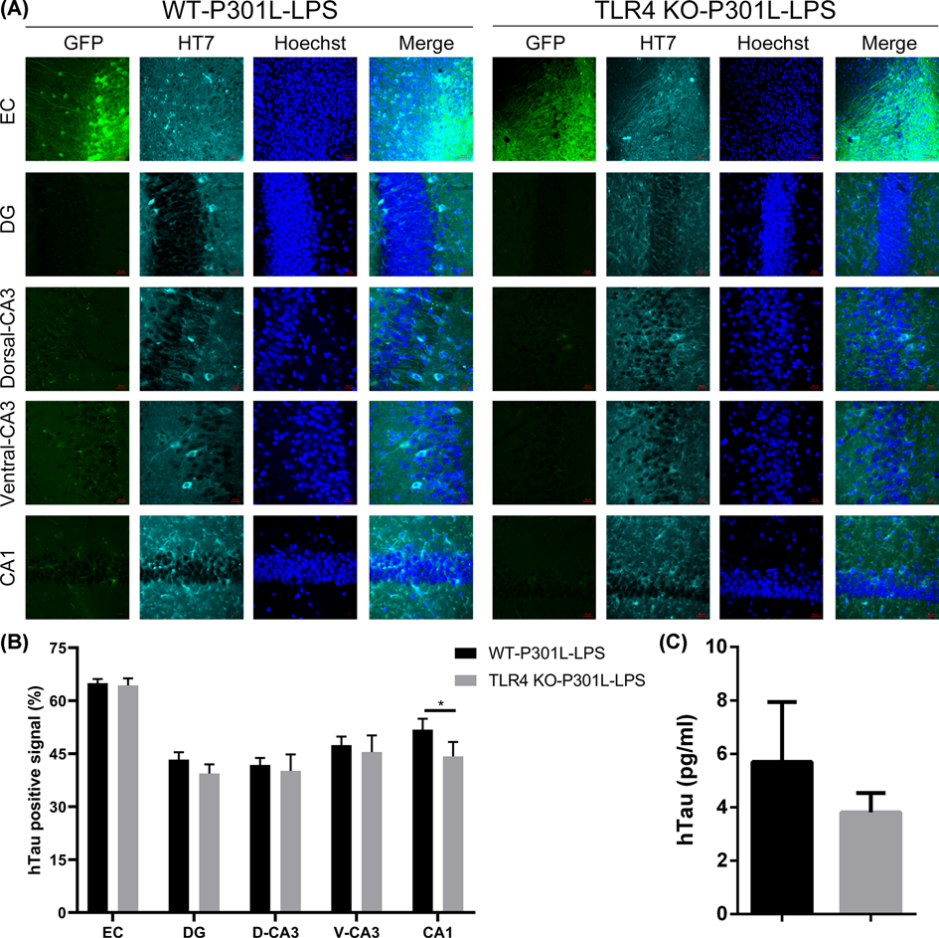
Knockout TLR4 does not ameliorate LPS-enhanced hTau transmission (**A**) Mice were subjected to immunofluorescence for GFP (green), HT7 (cyan), and Hoechst (blue) in the EC, DG, CA3 and CA1. EC scale bar, 50 μm; DG, CA3 and CA1 scale bar, 20 μm. (**B**) Quantification of hTau positive signal (HT7) in EC, DG, CA3 and CA1. Data were presented as mean ± SD (*n* = 3 each group, two-way ANOVA followed by Tukey *post hoc* test). *, *P* < 0.1. (**C**) TLR4 KO decreased interstitial fluid hTau level in DG subset collected by microdialysis, but the reduction was not statistically significant measured by ELISA. Data were presented as mean ± SEM (*n* = 6, each group, Student’s unpaired *t*-test).

By using HT7 to probe hTau, we detected hTau spreading from EMC to hippocampal DG, CA3 (both dorsal and ventral) and CA1 subsets in WT-P301L-LPS group mice, while TLR4 knockout only slightly attenuated the hTau spreading (Figure 6A,B). By microdialysis at DG subset and ELISA assay, a decreased hTau level in TLR4 KO-P301L-LPS group was detected but the reduction was not statistically significant when compared with the control group (Figure 6C). These data suggest that TLR4 KO knockout cannot prevent LPS-enhanced hTau transmission, suggesting the involvement of a non-microglia-dependent pathway in LPS-enhanced hTau transmission.

### Impaired BBB is involved in LPS-enhanced hTau spreading and GC preserves the integrity of BBB

**Figure 7:**
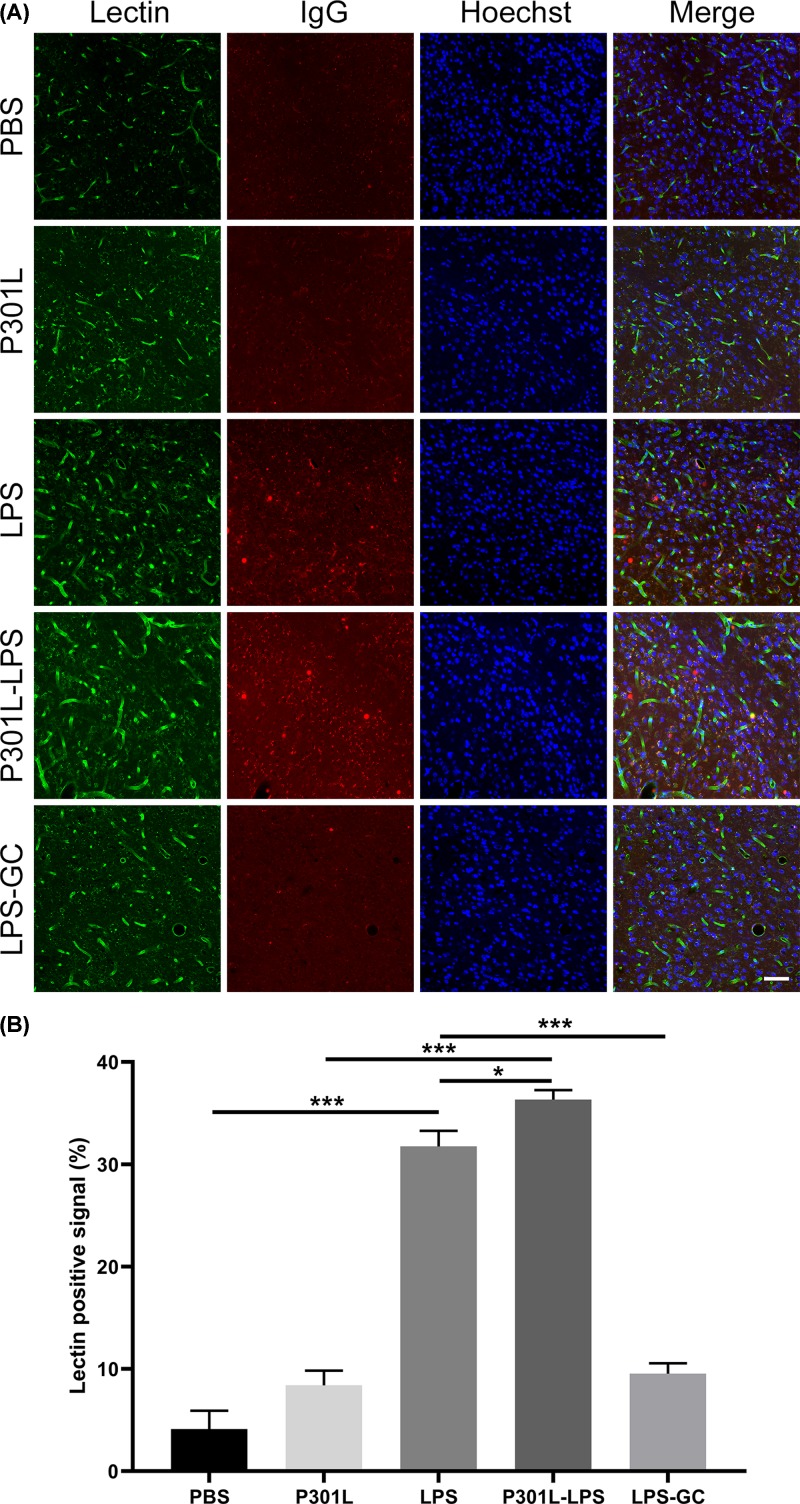
LPS disrupts integrity of BBB and GC treatment preserves the integrity (**A**) Mice were injected through peritoneal with LPS or PBS for 5 weeks, and then hippocampal slices were prepared. The blood vessels, the integrity of BBB and the nuclei were detected by co-immunofluorescence staining using lectin (green), IgG (red), and Hoechst (blue), respectively; scale bar, 50 μm. (**B**) Quantification of lectin positive signal showed a significantly increased leak of IgG from blood vessels in LPS and P301L-hTau-LPS groups. Data were present as mean ± SD (*n* = 3 each group, one-way ANOVA followed by Tukey *post hoc* test). *, *P* < 0.1; ***, *P* < 0.001.

As an inducer of neuroinflammation, LPS not only activates microglial cells, especially microglia in the mouse brain, but also disrupts the integrity of the BBB. Therefore, we measured the integrity of BBB by immunofluorescence staining to detect the leakage of indigenous mouse IgG into the perivascular spaces from blood vessels by using lectin. A significantly increased leak of IgG from blood vessels was detected in LPS and P301L-hTau-LPS groups compared with P301L-hTau groups, and GC attenuated LPS-induced IgG leakage (Figure 7A,B). To explore the mechanisms by which GC attenuated LPS-induced BBB leakage, we detected intercellular cell adhesion molecule-1 (ICAM-1) and tight junction proteins (such as Occludin and ZO-1) in the hippocampus. We found that Occludin and ZO-1 significantly decreased, and ICAM-1 significantly increased in P301L-hTau-LPS group compared with P301L-hTau-PBS group. GC could attenuate LPS-induced inflammatory response and the damages of BBB tightness (Supplementary Figure S3). These data suggest that an impaired BBB may be involved in the LPS-enhanced hTau spreading and GC treatment attenuates hTau spreading by protecting the integrity of BBB.

**Figure 8:**
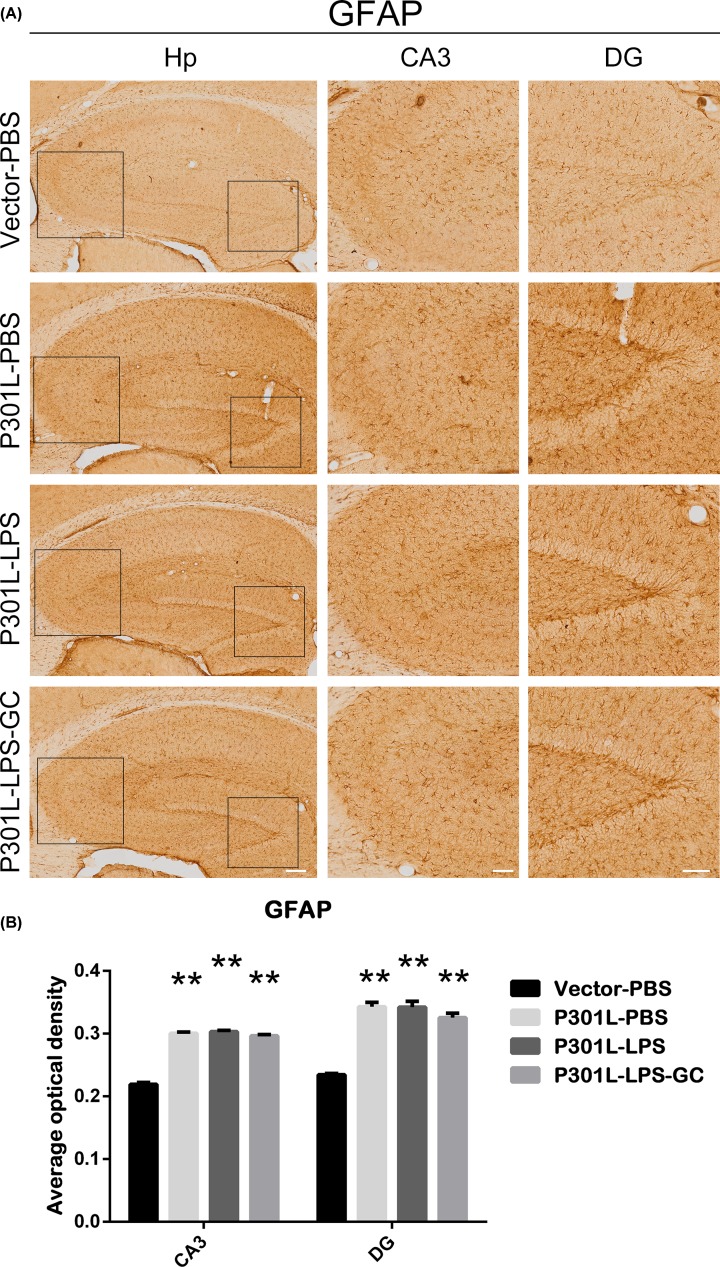
LPS does not enhance P301L-mediated astrocytes activation in DG subset, and addition of GC did not attenuate astrocyte activation (**A**) Activation of astrocytes (GFAP) was measured by immunohistochemical staining. Hippocampus (Hp) scale bar, 200 μm; CA3 and DG scale bar, 100 μm. (**B**) Average optical density (AOD) was used to analyze the activation of astrocytes. Data were present as mean ± SD (*n* = 3 each group, two-way ANOVA). **, *P* < 0.01 versus Vector-PBS.

Astrocyte is an important component of BBB, therefore, we further detected whether astrocyte is involved in LPS-induced impairment of BBB. The results showed that overexpressing P301L-hTau significantly activated astrocytes (GFAP), but LPS did not induce further activation of astrocytes (Figure 8A,B). Furthermore, GC treatment did not attenuate LPS-induced astrocytes activation in DG subset (Figure 8A,B). These data suggest that astrocytes may not be involved in LPS–BBB impairment.

## Discussion

The neurofibrillary tangle formed by the aggregated tau proteins is an important pathological features of AD, and the degree was positively correlated to cognitive decline [19]. In the early stages of AD, tau pathology first appeared in EC and then spread to the hippocampus. Many researchers had focused on the mechanism of tau transmission. It was shown that tau transmission depends on trans-synaptic pathways including three patterns, i.e. synaptic activity-, exosomes- and tunnel nanotubes-dependent transmissions [20]. It was also reported that clearance of microglia and inhibition of exosome synthesis could halt tau transmission in P301L mouse model [8]. Destruction of BBB integrity has already appeared in the early stage of AD [21], which plays an important role in the inflammatory response during AD [22,23]. An impaired BBB was involved in the formation of β-amyloid lesions and neurofibrillary tangles in the brain [24]. However, it is not reported whether BBB affects tau transmission. Here, we constructed a rapid hTau overexpression model in mice by injecting human mutant P301L-hTau in mouse MEC [16], and then injected LPS, a common neuroinflammation inducer to destroy the integrity of BBB, through intraperitoneal of the mice. Our results indicate that BBB leakage is involved in LPS-induced hTau transmission from entorhinal cortex to hippocampus, though a causal relationship between BBB linkage and hTau transmission needs further investigation. In addition to the BBB linkage, acute microglial activation can also contributes to the hTau transmission as observed in the current paper and a previous study [8], although inhibiting microglia did not significantly attenuate the chronic LPS-induced hTau transmission.

As a most widely used and effective anti-inflammatory agent in the clinic, another very important role of GC was to repair the BBB integrity [25], therefore we used GC as an antagonist of LPS. The behavioral detection in mice revealed a decrease in cognitive function in the P301L-PBS group compared with the Vector-PBS group, but it was not significant. Compared with the control groups, both depression-like behaviors and memory impairments were significantly shown in P301L-LPS mice. After GC intervention, cognitive impairment was improved. In AD mouse models, tau pathologies in hippocampus were first shown in DG and CA3 subsets [26,27], which are the important regions in maintaining spatial learning and memory. Using immunofluorescence to detect the expression of hTau in DG and CA3, we found many hTau positive signals in P301L-LPS group, whereas no expression of hTau was found in P301L-PBS group. At the same time, tau transmission was significantly reduced by GC intervention. These data suggest that LPS accelerates tau transmission from EC to DG and CA3, and GC intervention can significantly inhibit LPS-induced tau transmission.

Toll-like receptor 4 (TLR4), a member of the Toll-like receptor family, is a transmembrane protein encoded by the TLR4 gene. TLR4 belonging to the pattern recognition receptor (PRR) family is the most important receptor for LPS-activated microglia [28–31]. Depletion of microglia halted tau transmission in AD mouse model [8]. To investigate whether LPS-induced tau transmission involves in microglia activation, we applied TLR4 knockout mouse model, in which microglia cannot be activated by LPS. We examined hTau level in DG, dorsal CA3, ventral CA3, and CA1 regions of the hippocampus after MEC overexpression, and found that TLR4 KO did not significantly attenuate LPS-induced hTau transmission when compared with TLR WT group. The hTau level in DG interstitial fluid collected by microdialysis and measured by ELISA also showed the similar results. Then, we examined the direct effect of LPS and GC on the BBB, the results showed that chronic LPS injection through intraperitoneal disrupted the integrity of BBB, and simultaneous GC intervention preserved the BBB. We further demonstrated that GC may attenuate LPS-induced BBB leakage by inhibiting vascular inflammation and enhancing BBB tightness. These data suggest that disruption of BBB integrity may be another pathway for LPS-promoted tau transmission. Interestingly, we also observed that microglia were remarkably activated by expressing P301L-hTau, but LPS did not further activated microglia. It is indicated that microglia activation might be already reached the threshold by P301L-hTau. Considering LPS did not significantly activate microglia but it induced P301L-hTau transmission, we speculate that LPS-induced BBB disruption, a non-microglia-dependent pathway, may play a more important role in P301L-hTau transmission than microglia activation in this model.

In conclusion, we find in the present study that both acute and chronic periphery inflammation induced by LPS accelerates hTau spreading from MEC to hippocampus, and the mechanism involves BBB impairment.

## Supplementary Material

Supplementary Figures S1-S3

## Abbreviations

Aβ: amyloid β
AD: Alzheimer’s disease
BBB: blood–brain barrier
DG: dentate gyrus
EC: entorhinal cortex
EPM: elevated plus maze
GC: glucocorticoid
LPS: lipopolysaccharide
MAP: microtubule-associated protein
MEC: medial entorhinal cortex
MWM: Morris water maze
NOR: novel object recognition
TLR4: Toll-like receptor 4
