# Supplementary material for: Peripheral inflammation promotes brain tau transmission via disrupting blood–brain barrier
Source: Biosci Rep. 2020 Feb 20;40(2):BSR20193629. doi: 10.1042/BSR20193629 (PMC7033313; doi:10.1042/BSR20193629)
Supplement: Supplementary Figures S1-S3 [file BSR-2019-3629_supp.pdf]

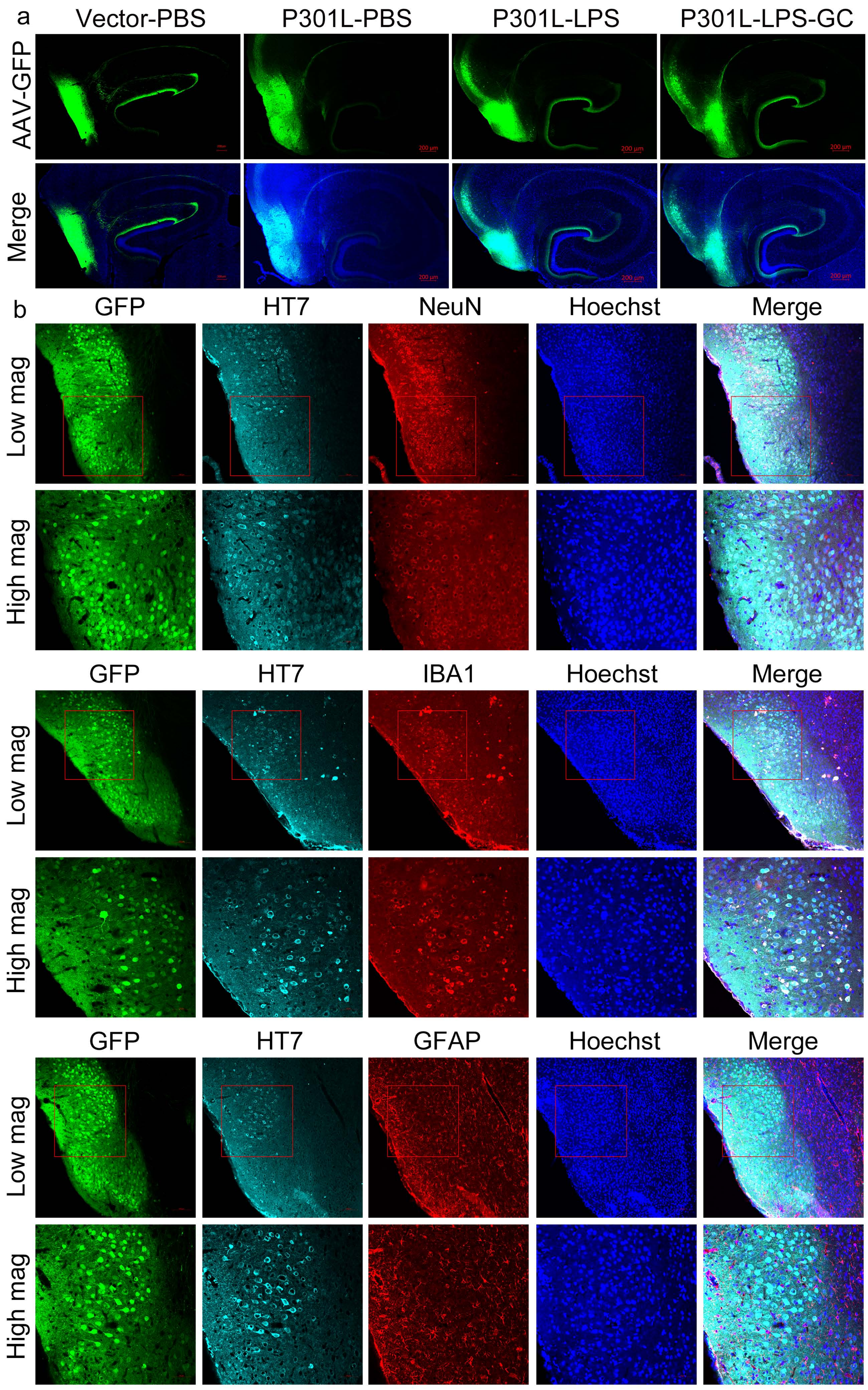
a
Vector-PBS
P301L-PBS
P301L-LPS
P301L-LPS-GC
AAV-GFP
Merge
200 μm
b
GFP
HT7
NeuN
Hoechst
Merge
Low mag
High mag
GFP
HT7
IBA1
Hoechst
Merge
Low mag
High mag
GFP
HT7
GFAP
Hoechst
Merge
Low mag
High mag

Supplementary Figure S1. Images showed the location, where the AAV-expressed tau was none-selectively detected, for each injection across the groups. (a) The initial site of injection was showed at low enough magnification by immunofluorescence staining using GFP (green) and Hoechst (blue). (b) The AAV-expressed tau was none-selectively detected in the EC subset by co-immunofluorescence staining of HT7 (cyan) and NeuN (red), or HT7 (cyan) and IBA1 (red), or HT7(cyan) and GFAP (red).

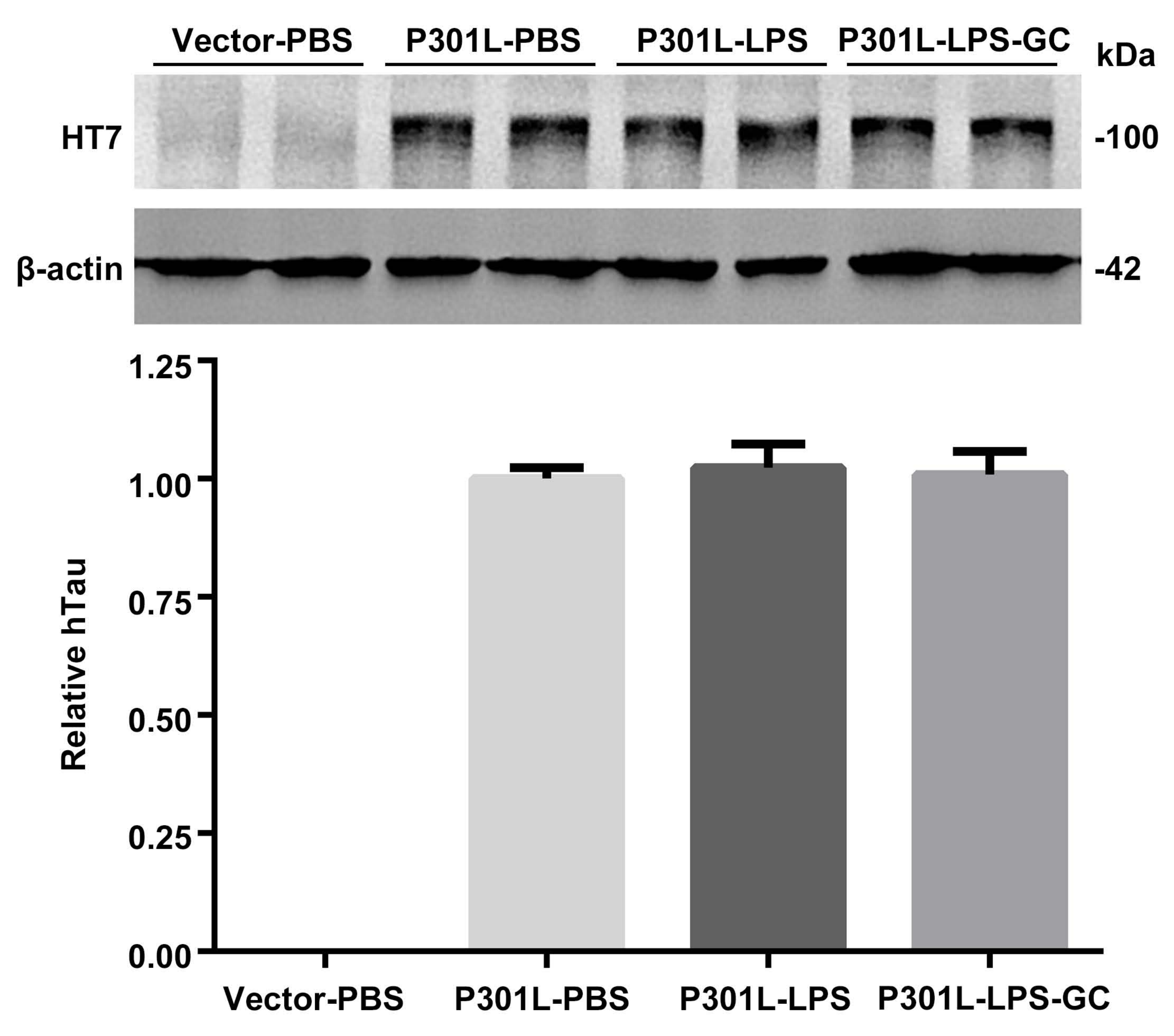

Vector-PBS
P301L-PBS
P301L-LPS
P301L-LPS-GC
kDa
HT7
-100
β-actin
-42
1.25
1.00
0.75
0.50
0.25
0.00
Relative hTau
Vector-PBS
P301L-PBS
P301L-LPS
P301L-LPS-GC

Supplementary Figure S2. Similar level of hTau was detected in EC subset in the three P301L groups. Brain tissue lysates in the EC subset were detected by Western blotting. Data were presented as mean ± SD (n=4 each group, one-way ANOVA followed by Tukey *post hoc* test).

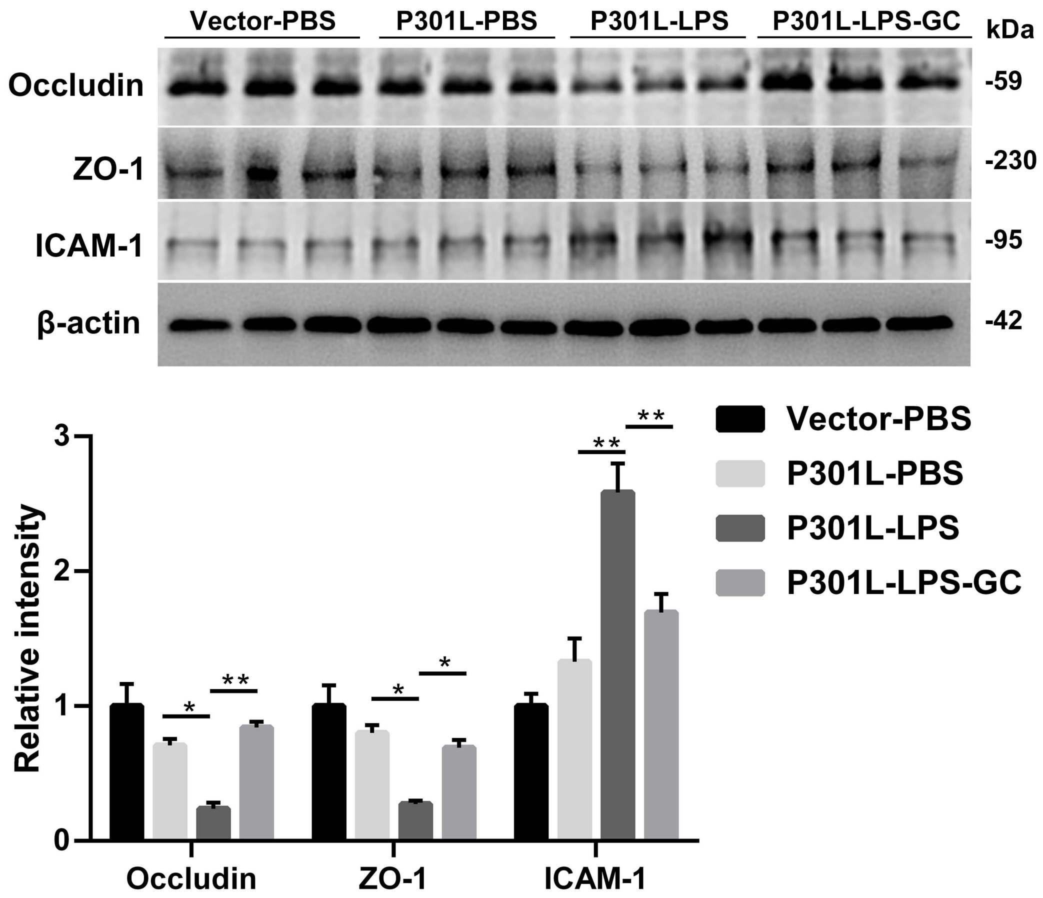
Vector-PBS
P301L-PBS
P301L-LPS
P301L-LPS-GC
kDa
Occludin
-59
ZO-1
-230
ICAM-1
-95
β-actin
-42
Relative intensity
3
2
1
0
*
**
*
*
**
**
Occludin
ZO-1
ICAM-1
Vector-PBS
P301L-PBS
P301L-LPS
P301L-LPS-GC

Supplementary Figure S3. Occludin, ZO-1 and ICAM-1 were detected in the hippocampus in the four groups. Brain tissue lysates in the hippocampus were detected by Western blotting. Data were presented as mean ± SEM (n=3 each group, two-way ANOVA followed by Tukey *post hoc* test). *, $P<0.05$; **, $P<0.01$.
